# An Exploratory Mixed Method Study for Developing and Psychometric Properties of the Sexual Information, Motivation and Behavioral Skills Scale (SIMBS) in Iranian Couples

**Published:** 2019-06-18

**Authors:** Fahimeh Bagheri, Effat Merghati Khoei, Majid Barati, Alireza Soltanian, Manoj Sharma, Reza Khadivi, Ali Ghaleiha, Vinayak K. Nahar, Babak Moeini

**Affiliations:** ^1^Department of Public Health, School of Public Health, Hamadan University of Medical Sciences, Hamadan, Iran; ^2^The Iranian National Center for Addiction Studies (INCAS), Tehran University of Medical Sciences, Tehran, Iran; ^3^Family-Sexual Health Division in the Brain & Spinal Cord Injury Research Center (BASIR), Neuroscience Institution, Tehran University of Medical Sciences, Tehran, Iran; ^4^Social Determinants of Health Research Center & Department of Public Health, School of Public Health, Hamadan University of Medical Sciences, Hamadan, Iran; ^5^Modeling of Noncommunicable Diseases Research Center , School of Public Health, Hamadan University of Medical Sciences , Hamadan, Iran; ^6^Department of Behavioral & Environmental Health, School of Public Health, Jackson State University, Jackson, Mississippi , USA; President, Health for All, Inc, Omaha, Nebraska, USA; ^7^Department of Community Medicine, Medical School, Isfahan University of Medical Sciences, Isfahan, Iran; ^8^Department of Psychiatry, School of Medicine, Hamadan University of Medical Sciences, Hamadan, Iran; ^9^Center for Animal and Human Health in Appalachia, College of Veterinary Medicine, DeBusk College of Osteopathic Medicine, and School of Mathematics and Sciences, Lincoln Memorial University, Harrogate, TN, USA; ^10^Department of Dermatology, University of Mississippi Medical Center, Jackson, MS, USA

**Keywords:** Validation studies, Spouses, Qualitative research, Behavioral sciences, Iran

## Abstract

**Background:** This study was designed to construct and assay the psychometric properties of a scale in order to recognize sexual information, motivation and behavioral skills of Iranian couples.

**Study design:** a mixed method study.

**Methods:** This was an exploratory mixed method investigation conducted in two stages from Sep 2017 to Jun 2018 in Isfahan, Iran. First, qualitative methods (individual interviews with 22 couples) were applied to generate items and develop the questionnaire. Second, psychometric properties of the questionnaire were assessed. Reliability was evaluated by composite reliability, Interclass Correlation Coefficient (ICC) and internal consistency. Moreover, Exploratory Factor Analyses (EFA) and Confirmatory Factor Analyses (CFA) were carried out to examine construct validity. To evaluate content validity were performed CVI and CVR.

**Results:** An item pool comprising 107 statements related to couple 'sexual information, motivation and behavioral skills were generated in the first stage. In the second stage, item reduction was exerted and the final issue of the questionnaire including 51 items was expanded. The evaluation of the psychometric properties of the final version displayed that the scale had good reliability and structure. The results from exploratory factory analysis demonstrated a 9-factor solution for the scale that jointly reported for the 39.5% of the observed variance. The mean scores of the CVI and CVR were 0.92 and 0.90, respectively. Additional analyses indicated acceptable results for composite reliability for the subscale of instrument ranging from 0.78 to 0.95.

**Conclusion:** The sexual information, motivation and behavioral skills scale is a reliable and valid instrument that can be used in future studies on Iranian couples.

## Introduction

Sexual health requires a positive and respectful approach to sex and sexual interactions and enjoyable sexual experiences free of coercion, discrimination and violence ^1^. However, in spite of these frequent emphasis and the basic significance of sexual health and sexuality for the general health and wellbeing of individuals and populations, the continued stigmatized of this view in intergovernmental dialogues negatively affects the access and use of sexual health services and programs^2^. In Iran, as one of the traditional-religious communities, the sex-based approach does not set a clear path for the sexuality of individuals, and sexual issues are always in a state of ambiguity^3^, whereas sexual behavior of people is related to how they get their sexual socialization. Sexual socialization refers to the process of development of gender identity and sexuality, the acquisition of information and skills, and finally the formation of sexual attitude^4^.

**Figure 1:**
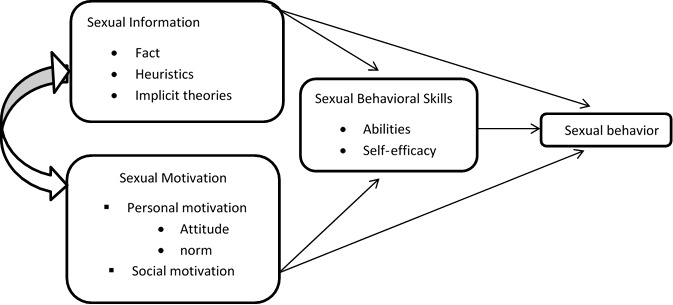
Information-motivation-behavioral skills (IMB) model

Regarding to determinant role of information, motivation and behavioral skills in promoting sexual behaviors, the Information, Motivation, Behavioral Skills (IMB) model was used as the main theoretical framework of this study. The IMB model is a popular social psychological conceptualization for understanding and promoting health-related behavior^5^. Empirical evidence confirms the effectiveness of this model in sexual health ^5, 6^. According to IMB model, if couples are well informed, well-motivated to perform sexual health behaviors, and obtain the sexual behavioral skills to act effectively, they will be more expert at acceding positive health outcomes such as sexual satisfaction ^5^(Figure 1). Since in the Iranian society there is little education in the field of sexual issues, and the family and society as the two effective institutions in the process of individuals 'sexual socialization have a passive approach^3^, sexual information of couples does not just include facts, but a major part of sexual information of people is based on heuristics (simple rules which permit automatic and cognitively effortless –but often incorrect– decisions) and implicit theories (complicated sets of beliefs that require cognitive effort to process and which are also often incorrect), easily activated by individual social ecology. Motivational structures include individual (attitude and norm) and social motivation (social support). The social support of individuals from the institutions of society (families, schools, universities, religious and cultural institutions and etc.) is contributing to the formation of the proper sexual behavior of individuals^5^. The construct of sexual behavioral skills and sexual behavior of people is also heavily influenced by sexual socialization and sexual norms that govern society^7^.

Most sexuality measures are focused on quality of sexual life^8^, intimacy and sexual satisfaction^9^ and sexual knowledge and attitudes^10^. Many of these available measures are not appropriate for applying in the Iranian society and culture as the only legally and culturally accepted type of sexual interactions in Iran is heterosexual marital relationship^11^. On the other hand, many existing scales developed for the sexual issue in Iran such as dyadic sexual communication scale^12^, female sexual distress scale^13^, sexual satisfaction^14^, sexual adjustment questionnaire^15^,sexual self-concept questionnaire^16^only focus on the assessment of a single aspect of sexual behavior, and overlook other influential aspects, while some scholars suggested that for studying sexuality in young people we need to construct the tools that are able to predict sexual behaviors^17^.

Thus, we aimed to develop and examine the psychometric properties of a newly developed scale in order to identify sexual information, motivation, and behavioral skills of Iranian couples.

## Methods

The present study was an exploratory mixed method research to develop and validate a culturally appropriate and meaningful instrument to assess couples' sexual information, motivation, and behavioral skills based on the same name IMB model. The study was performed in two stages. The literature review and a qualitative approach were applied in order to generate items and construct the questionnaire in the first stage. We assessed the Psychometric properties of the scale in the second stage.

### Stage 1: Item generation and scale development

In the qualitative research, individual interviews, was used to generate items and develop the scale. IMB model constructs were used as the theoretical basis for the development of scale.

### Participants and data collection

We recruited 22 couples (n=43) consisting of 22 women with their husbands (21 men; one man did not participate). Participants were new or recently married couples who lived in Isfahan City (the center of Iran). People married within past five years are called new couples^18^. Couples who volunteered to participate in the study ranged between the ages from 21 to 35 yr and varied in their educational levels, occupational backgrounds and socioeconomic status. For data gathering, in-depth, face-to-face, and semi-structured interviews were employed. Talking about sexuality in Iranian culture is very difficult and often associated with shame. Therefore, each interview started with a general and more well-known question about the participants' life history. Then the interviewer slowly moved to those aspects that more directly related to the interview guide such as how do you portray sexual interactions and their formation? After primary answers, participants were countenanced to narrate their sexual life stories. To facilitate the sessions a semi-structured inventory based on theoretical framework of study (IMB model) was used. Overall, 43 individual interviews were held with the participants and data saturation was attained after 40 individual Interview.

### Data analysis

Qualitative content analysis were employed inspired by Graneheim and Lundman’s approach. In this approach ‘whole interviews or observational’ protocols is the most suitable unit of analysis^19^. Data analysis process was done concurrently and continuously with data collection. Each individual interview was transcribed verbatim and analyzed before the next interview to happen. We arrived a total understanding of the data, by reviewing each transcribed text several times. Then, the units of meanings were elicited from the testimonies. This punctual unit was abstracted to a “condensed meaningful unit”. Codes were elicited based on these units. We used line by line encoding for data analysis. Codes were formed during frequent discussions between researchers. The elicited meanings were used for item generation. Items were modified and reduced based on the expertise valuation.

### Stage 2: Psychometric assessment

The pre final draft included 107 items that measured the constructs of IMB model. Sexual information was measured by 27 items, for example "for having a good sex, one's thoughts should be focused on the relationship". Sexual motivation was examined by 50 questions, such as "I'm getting into sex for peace of myself and my wife/ husband". Sexual self-efficacy was evaluated with 11 questions, such as "I can demand sex from my wife/ husband when needed" and sexual behavior was examined by 19 questions, for example "I talk to my wife/husband about the proper time to have sex". The items were rated on a 5-point Likert response format scale ranging from strongly agree=5 to strongly disagree=1. This pre final draft has been used for psychometric evaluation.

### Participants and procedures

This cross-sectional validation study was performed on 200 new couples (n=200 women; n=200 men) from Sep 2017 to Jun 2018 at Isfahan, Iran. Multistage random sampling method was used for selecting participants that included clustering and simple sampling. At the first step, cluster sampling was executed. The list of health centers in the health deputy of Isfahan Province was used as a statistical framework for determining the clusters. We selected 20 health centers in different sections of the city among 81 health centers of Isfahan city based on grouping the health department of the Isfahan Province from the covered health centers by the indicators of health services development in the country. In the second step, 20 eligible couples from each of the 20 selected health centers were picked out by simple sampling method. The inclusion criteria were 1) Having five years or less marriage history, 2) Participate both women and husband in the study, 3) not having any under treated mental illness, 4) not having severe marital conflicts, 5) residing in Isfahan city, Iran and 6) willingness to participate in the study. The trained health care providers in each health center facilitated access to couples and did the sampling. They selected eligible couples and explained the study objectives and the questionnaire completion style for them. After giving written consent from the subjects, the questionnaire was given to them. The individuals completed anonymous questionnaires at home. After completion, the couple put the questionnaires in a folder and left it at a determined place in the health center to preserve more privacy.

### Statistical analysis

***Validity:*** We calculated face, content and construct validity of Sexual Information, Motivation and Behavioral Skills Scale (SIMBS) in couples as follows:

***Face validity:*** Face validity was applied in two stages (qualitative and quantitative). In the Qualitative stage, five couples were demanded to evaluate the questionnaire to assure that participants understood questions and had no ambiguity or difficulties in responding to the questionnaire. Impact score (frequency × importance) was calculated in the quantitative phase to demonstrate the proportion of participants who recognized the question was important or quite important. If items had an impact score equal to or greater than 1.5 were considered as appropriate (which corresponds to a mean frequency of 50% and a mean importance of 3 on the 5-point Likert response format scale)^20^.

### Content validity

Content validity was done to confirm that the scale comprehensively covers domains of interest and was culturally relevant and clear. We applied qualitative and quantitative content validity. An expert panel including 14 scholars specialized in sexuality, health education, and psychologists appraised the content validity of the questionnaire. In the qualitative stage, they assessed wording, grammar, item allocation and scaling of the questionnaire. Items were modified based on specialists' comments. The quantitative stage was conducted to calculate the content validity index (CVI) and the content validity ratio (CVR). Relevancy, simplicity and clarity of each question are estimated by CVI measurement ^21^. To assess the CVI, a Likert-type, ordinal scale with four possible responses was used. The responses include a rating from 1= not simple, not clear and not relevant, to 4= very simple, very clear and very relevant. The CVI for every question was computed using following formula

$$CVI=\frac{The\;number\;of\;panelists\;specifying\;the\;question\;as\;simple\;clear\;or\;relevant(rating\;3\;or\;4)}{total\;number\;of\;content\;experts}$$

The acceptable lower limit for CVI value is considered 0.79 ^22^. The essentiality of each question for the Iranian culture examined by CVR using 3-points rating scale (essential, useful but not essential, and not essential). The CVR for every question was computed using formula CVR = [Ne − (N/2)] ÷ (N/2) (Ne is the number of panelists indicating "essential" for each special question and N is the total number of panelists). The numeric value of CVR is specified by Lawshe Table. An acceptable CVR value for 14 panelists is 0.51, According to the Lawshe Table ^23^.

### Construct validity

The Exploratory Factor Analysis (EFA) was conducted to specify the dimensionality of the scale (or structure detection). The EFA examined the underlying relationships between the variables. Maximum likelihood extraction method and Varimax Rotation Kaiser Normalization were performed. Factor loading equal to or greater than 0.4 was considered acceptable which indicates an important relationship between items and factors ^24^. We used Kaiser-Meyer-Olkin (KMO) Measure of Sampling Adequacy and Bartlett test to appraise sampling adequacy to execute a satisfactory factor analysis. If the value of KMO is greater than 0.7, then correlations between the data will be suitable for factor analysis. The criteria of minimum eigenvalues >1 (Kaiser Criterion) were used to determine the subscales (factors).

We also conducted confirmatory factor analysis (CFA) with 100 new couples (n=200) to evaluate how well the model elicited by EFA and the theoretical framework behind the scale fitted the observed data. Due to the large number of questions, Smart PLS 2.0 software was used for the CFA. We assessed the construct validity and reliability through a number of indices such as factor loadings, cross-loadings, average variance extracted (AVE), Cronbach’s alpha and composite reliability. The convergent and discriminant validity of the questionnaire was examined. Convergent validity in PLS for reflective constructs is supported if the average variance explained (AVE) for each construct is 0.5 or greater and if each item’s loading on its assigned construct is significant as determined by the t-value. Discriminant validity is confirmed if the measures of constructs that should not be theoretically relevant to each other are in fact observed not to be relevant to each other^25^. Finally, a goodness of fit (GoF) index was calculated to demonstrate the model fit to the data^26^. The results of the descriptive data were acquired with SPSS version 21 (Chicago, IL, USA).

### Reliability

Reliability of the scale items was evaluated via computing the Cronbach’s alpha coefficient and composite reliability. Acceptable composite reliability value is greater than 0.7; and a value below 0.6 represented loss of reliability. Moreover, the alpha values of 0.50 or above were considered satisfactory. The following class was choosing to explicate the contract levels: α≤0.5 was considered unacceptable, 0.50-0.60 poor, 0.60-0.70 moderate, 0.70-0.80 good, 0.80-0.9 very good and >0.90 excellent. Intraclass correlation coefficient (ICC) was computed for assessing questionnaire’s stability by conducting test-retest reliability. Ten couples (20 participants) completed the questionnaire twice with three-week intervals. The test-retest scores for each construct were compared using Pearson correlation test. ICC values of 0.40 or higher were considered satisfactory (r ≥ 0.81-1.0 as excellent, 0.61- 0.80 very good, 0.41-0.60 good, 0.21-0.40 fair, and 0.0-0.20 poor) ^27^.

### Ethical Considerations

The study was approved by the Ethics Committee of Hamadan University of Medical Sciences with proprietary ID, IR.UMSHA.REC.1395.435 and agreed by administrators at the research sites (Isfahan University of Medical Sciences). The couples were aware that participation in the study was absolutely optional, none of the participants would be recognized in any disseminations rising from the study and their confidentiality would be protected. All participants read the written consent carefully and signed it before entering the study.

## Results

In the second stage, 200 couples (n=200 women; n=200 men) were enrolled. The mean age of participants was 29.4±4.8 yr that duration of their marriage was 3.53 ± 1.4 year (Table 1).

The result of quantitative face validity showed that impact score for all items had ranged from 1.5 to 5; therefore, none of the items was deleted. Participants accounted small variations in the wording of some questions for more clarity in the qualitative face validity.

Questions with CVR and CVI less than 51 and 79, respectively, were deleted (14 questions), in the quantitative content validity stage. Wording, grammar, and item allocation were corrected according to the experts’ viewpoints in the qualitative stage. For example, the sentence " In my sexual life, the initiation of sex must be from my husband" changed to "In my sexual life, my husband should start the relationship" or the " Men and women should be similar in sexual desire" was corrected to " Sexual desire of men and women should be compatible with each other". Moreover, the quantitative content validity result displayed that the mean scores of CVI and CVR were 0.92 and 0.90; respectively.

We used the exploratory factor analysis (EFA) to measure construct validity. The Kaiser-Meyer-Olkin (KMO) and Bartlett’s test demonstrated that the data was adequate for factor analysis (KMO index=0.753, χ2=15261.69, *P*=0.000). Maximum likelihood analysis with varimax rotation recognized nine factors (self-efficacy, attitude, fact, skill, behavior, social support, implicit theory, heuristic and norm) with eigenvalues greater than 1and factor loading equal to or greater than 0.4; accounting for 5.39% of variance observed and the highest expressed changes were related to the Attitude.

**Table 1:** Demographic characteristics of the study sample (Stage 2, n=400)

**Variables**	**Number**	**Percent**
Educational status		
Under the diploma	28	7.0
Diploma	120	30.0
Bachelor	200	50.0
Masters	42	10.5
PhD	10	2.5
Occupational status		
Employee	131	32.7
Worker	31	7.7
Free job	99	24.7
Housewife	132	33.0
Non-response	7	1.9
Marriage style		
Traditional marriage	318	79.5
Modern marriage	79	19.8
Non-response	3	0.7
Family marriage		
Yes	110	27.5
No	290	72.5
Number of children		
0	156	39.0
1	196	49.0
2	45	11.3
3	3	0.7

The values for the items of all constructs overpassed the threshold of convergent validity (Table 2). Discriminant validity of the measures was specified by ascertaining that the square root of the AVE of each construct is larger than its correlations with other constructs approved for all factors except the fact (Table 3).

**Table 2:** Descriptive statistics of nine dimensions of Sexual Information, Motivation and Behavioral Skills Scale (SIMBS) and Results of Outer Model Analysis (Reliability and Validity Tests)

**Variables**	**Males**		**Females**		**ICC**	**Composite** **Reliability**	**AVE**
	**Mean**	**SD**	**Mean**	**SD**			
Abilities	8.8	3.7	8.7	3.9	0.613	0.837	0.566
Attitude	41.8	5.6	42.5	4.8	0.750	0.951	0.624
Behavior	19.4	4.4	19.5	3.6	0.450	0.883	0.521
Fact	21.1	3.8	21.4	3.5	0.527	0.898	0.560
Heuristics	8.8	1.9	8.9	1.9	0.721	0.843	0.728
Implicit Theory	6.6	2.1	6.9	2.3	0.713	0.805	0.580
Norm	8.5	2.6	9.1	2.1	0.598	0.785	0.549
Self-Efficacy	34.3	6.8	34.4	6.4	0.658	0.952	0.649
Social motivation	2.9	2.1	3.4	2.2	0.796	0.915	0.843

**Table 3:** Discriminant Validity (Square Root of AVE)

**Variable**	**Abilities**	**Attitude**	**Behavior**	**Fact**	**Heuristics**	**Implicit Theory**	**Norm**	**Self-Efficacy**	**Social motivation**
**Abilities**	0.752								
**Attitude**	0.281	0.790							
**Behavior**	0.324	0.667	0.722						
**Fact**	0.228	0.781	0.594	0.748					
**Heuristics**	0.299	0.575	0.476	0.573	0.853				
**Implicit Theory**	0.164	0.394	0.403	0.450	0.438	0.761			
**Norm**	0.288	0.599	0.569	0.593	0.534	0.442	0.741		
**Self-Efficacy**	0.283	0.673	0.717	0.539	0.449	0.317	0.492	0.805	
**Social motivation**	0.214	0.074	0.183	0.176	0.186	0.326	0.097	0.176	0.918

All of the indicators had acceptable composite reliability ranged from 0.78 to 0.95. The Cronbach’s alpha coefficient used for evaluating internal consistency of subscales of questionnaire ranged from 0.59 to 0.94 which indicates the moderate to excellent internal consistency. In addition, the ICC for the questionnaire subscales was computed, which ranged from 0.45-0.79 (acceptable) lending support to the consistency of the questionnaire (Table 2). Finally, GoF=0.72, indicating the model had good fit.

## Discussion

The purpose of this study was to develop and execute validity and reliability trying of the Sexual Information, Motivation and Behavioral Skills Scale (SIMBS) in newly married Iranian couples. The IMB model is one of the theoretical models used in explaining sexual health promoting behaviors^28^. The IMB model is the parsimonious model that prepares a viable framework for creating health promotion interventions, its constructs are operationally specified, and it ascertained the causal relationship between its theoretical determinants^5^. According to the authors' knowledge, to date, no study has been found to specify whether the IMB model accounts for promoting sexual health in couples In Iran to compare the results of the present study.

The results of psychometric indices on the SIMBS indicated that the scale has a good validity and can be a primary tool for measuring the structures of the IMB model. Rigorous and appropriate processes were used to validate SIMBS. The SIMBS was developed based on explained concepts in the qualitative stage of the study done based on the IMB model with 107 items. Content validity executed in this study was led to remove 14 questions from the primary questionnaire. An exploratory factor analysis with maximum likelihood was performed on a pre-final form with 93 items and 23 items were removed at this stage. The questionnaire with 70 questions was classified in nine categories including fact; heuristics, implicit theory, attitude, norm, social motivation, abilities, self-efficacy and behavior. The results of the analysis due to the KMO index portend sufficient sample size and desired factor analysis.

Nine factors recognized in this study described 39.5% of the variance observed. Factors 2 and 3 (attitude and fact) contributed the most to the total variance 11.35 and 7.9, respectively. The major role of the two factors of attitude and fact in the overall variance is justified by the fact that the individual's behavior is a function of his knowledge and attitude^29^. In other words, the healthy and proper sexual behavior of a person is based on his beliefs and knowledge. In consistent with our findings, the IMB model explained 41% of variance of diabetes medication adherence in a sample of adults with type 2 diabetes^30^. The IMB model expressed 29.8% of the variance in healthy behaviors of adults with metabolic syndrome^31^. The IMB constructs described 37.9% of the variance in adherence in breast cancer patients^32^. The scale was evaluated using CFA. CFA can provide more exact and decisive evaluation of latent factors and appraise goodness of fit results of factor structure of a questionnaire^33^. In the CFA stage 19 items were removed and final scale with 51 items was presented. The final model had a good fit (0.72). Moreover, convergent and divergent validity represented satisfactory results for all factor and only divergent validity for the fact was not completely approved that may be due to the low loading of one of the questions in this factor. Future work on this scale can look into clarifying this item further. Moreover, the composite reliability, intraclass correlation coefficient and Cronbach’s alpha coefficient were admissible and displayed good reliability and stability for the scale.

Along with our results, in the study on outpatients in Anhui second-level hospitals, the internal consistency of the constructs of the IMB was announced between 0.71 and 0.92^34^. There are a few questionnaires that measure sexual knowledge and attitudes ^35, 36^ of Iranian couples that their psychometric properties have reviewed and approved. However, the information structure in the IMB model, moreover to the facts that is equivalent to knowledge, contains two other subscale heuristics which expresses learning based on experiences, especially trial and error and implicit theory that review the mental structures of couples in the field of sex^28^. Considering the conservative approach of Iranian society in the field of sexual issues and the lack of formal education for people in this field until marriage^37^, examination heuristics and implicit theories, is necessary.

Although the present study has several strengths, it has some limitations. Sexuality is a relatively private issue with varying degrees of social, cultural, moral, religious and legal restrictions and norms^38^. Research on sexual issue faces with difficulty in Iran because talking couples about the most private issues of their lives is shameful and fearful of disclosure. Thus, we tried to decrease this limitation by building trust through explaining the goals and stages of the study clearly and in detail, anonymity and confidentiality of the questionnaire, filling the questionnaires by the couples at their home and putting completed questionnaires at a specific location at the health center. The studied population were couples living in Isfahan city. In spite of the researchers' efforts to select couples with the greatest diversity in demographic characteristics, more than half of them had high school baccalaureate which can form an important confound factor for conclusions about the comprehension and stability of the scale; so further studies in other geographic locations or at other facilities required to approve the applicability of the SIMBS in Iranian couples.

## Conclusion

The scale displayed good construct validity and all subscales demonstrated high internal consistency and acceptable composite reliability. The Sexual Information, motivation, Behavioral Skills Scale (SIMBS) in Iranian couples is a valid and reliable instrument. In addition, further researches are counseled to comprehend the weaknesses and strengths of the scale when it is applied for other population in other contexts.

## Acknowledgements

This paper has been extracted from Fahimeh Bagheri’s PhD thesis in Health education and health promotion. The author's special thanks go to the couples who participated in this study. Thanks to Hamadan University of Medical Sciences for supporting the research.

## Conflict of interest

The authors declare that they have no competing interests.

## Funding

This study was funded by Hamadan University of Medical Science under Grant No 9510286293.

## Highlights

- This scale measures the sexual information, motivation and behavioral skills (IMB) of Iranian couples.
- The CVI, CVR, convergent and divergent validity and reliability of scale were reviewed and approved.
- The final questionnaire consists of 51 questions that measure 9 substructures of IMB model.
- Nine factors recognized in this study described 39.5% of the sexual behavior variance.
- The IMB model is appropriate model for promoting sexual health in Iranian couples.
